# Lecture on the History of Discoveries Concerning the Circulation of the Blood

**Published:** 1882-05

**Authors:** Wm. Ruthmrford

**Affiliations:** Professor of Physiology


A LECTURE ON THE HISTORY OF DISCOVERIES CONCERNING THE CIRCULATION OF THE BLOOD. Introductory to the Course of Physiology. Delivered in the University of Edinburgh, October 28, 1881, by WM. RUTHMRFORD, M.D., F.R.S., Professor of Physiology.

GENTLEMEN:—It has for some years past unfortunately become customary on the part of certain opponents of medical progress, to systematically represent that nothing of any value has been learned by experiments on animals. These persons have been "heard because of their much speaking." By the frequent repetition of statements calculated to produce an erroneous impression upon those who have not been educated in biological science, they have succeeded in persuading many intelligent persons to believe their false report. It seems to me perfectly plain that we must not any longer merely trust the common sense of the public to perceive that they are being systematically misled by the enemies of science. We must trouble ourselves more than we have hitherto done to provide the public with a true account of the manner in which important physiological discoveries have been arrived at; and, as "truth is great and will ever prevail," I am sure that, if we put our case clearly, it will not fail to convince minds that are unprejudiced. I cannot in the course of an hour do more than allude to a limited part of physiology, and I have chosen the circulation of the blood, because that is a typical case, from which the others may be judged.

It was long a prevalent idea among the ancients that the blood of the body is contained in the veins and right side of the heart, while the left side of the heart and arteries are filled with air derived from the lungs and distributed to all parts of the body to keep it cool. So completely did this idea—of blood in the veins only—occupy the minds of classic writers, that even now, were any one on some rhetorical occasion to say, *e.g.*, that Mr. Gladstone has Scottish blood in his *arteries*, he would be regarded as an iconoclast of classical phraseology. That old erroneous idea resulted from lack of knowledge regarding the many differences that exist between a dead and a living body. It is true that the blood, to a large extent, leaves the arteries at death, and accumulates in the veins, and that when both sets of vessels are cut across after death, the thin-walled vein collapses, while the thick-walled artery remains patent and becomes filled with air by the elasticity of its wall. But it is equally true that until the artery is opened, there is no air in it, either during life or after death.

In the time of Galen it came to be recognized that the arteries contained blood, but it took many a long century after him to discover that the arteries contain blood without an admixture of air. Galen was a Roman of the second century. He was the physician and friend of the great Emperor Marcus Aurelius. He was a man of much force of character, and an indefatigable investigator. His book on the "Uses of the Parts of the Body" is of great historical interest to the physiologist. It shows that Galen was always in his researches actuated by the conviction that "nature does nothing in vain," and that every part of the body must have some utility. He dissected the dead, he experimented on living animals, and he watched the effects of diseased conditions which have, aptly enough, been designated the "experiments of nature." By observations made by these three methods, he criticized the ideas of his predecessors, and advanced many new doctrines, some near the mark, many wide of it. It is not surprising that many of his theories should have turned out erroneous, for with a mechanism like the animal body, so complicated and difficult of comprehension, how could he, in the infancy of science, do otherwise than err greatly on many points which chemistry and other developments of modern science were needed to explain?

Galen's teaching with regard to the movement of the blood deserves our attention, for it lasted from the second until the seventeenth century, when it received its final blow from Harvey. Galen said, since the different parts of the animal body require to be nourished, nature has provided the alimentary canal, a large channel common to all the nourishing materials, and a system of smaller channels—the vessels which convey the nutriment to all parts of the body. In the stomach the food undergoes a primary elaboration, and the material suitable for nourishment passes into the veins of the stomach and intestines, and thence by the vena porta to the liver, where it undergoes a second purification, the useless material being thrown away as the bile, and the remainder becoming "perfect blood." He compared the "liquid humor" found in the portal vein to the juice just expressed from the grape —which must undergo fermentation and refinement before it becomes real wine. To Galen, therefore, the liver was a blood-making organ; it was the "fountain of the blood," which flowed away by the hepatic veins to the vena cava, to be conveyed to all parts of the body (*lib. cit.*, bk. i., ch. v). According to Galen, the blood flows up the vena cava to the head, neck, and arms, and down that vessel to the lower parts of the body; and everywhere the tissues are nourished by blood brought to them by the veins. He regarded the right auricle of the heart as a sort of diverticulum from the vena cava, through which some of the blood passes from the vena cava into the right ventricle. From the right ventricle it takes two courses ; part of it passes through the pulmonary artery to nourish the lungs, while the remainder passes through pores in the septum of the heart into the left ventricle. There it is mingled with air drawn from the lungs through the pulmonary veins, and the mixture of blood and air leaves the heart by the aorta, to be distributed by the arteries to all the organs. So, said he, every organ is supplied with a vein, an artery, and a nerve; the blood in the vein nourishes it, the artery supplies it with vital spirits and keeps it cool, and the nerve gives it sensibility. Galen evidently thought that by getting the blood from the right to the left side of the heart through its partition wall, he had triumphantly explained how it is that in the living animal there is blood in the left as well as in the right ventricle of the heart, and yet he was still in harmony with the doctrine of his teachers, that air passes from the lungs to the heart and arteries through the pulmonary veins.

But he started another unfortunate error, which was even in the seventeenth century, supported by Riolan, of Paris, in opposition to Harvey. Galen stated that everywhere throughout the body the arteries and veins frequently anastomose by small openings, whereby an interchange of blood and air takes place (*lib. cit.* bk. vi. ch. x.) With that idea we must return for a moment to the lung. Galen made the discovery that the pulmonary veins are not merely air-tubes, as previously supposed, but that they contain blood as well as air, and he accounted for the presence of the blood by supposing that it escapes from the pulmonary artery through his imaginary anastomoses between it and the pulmonary vein. He said that the blood is squeezed through these minute openings by the contraction of the chest, "since the sigmoid valves at the orifice of the pulmonary artery prevent its reflux to the heart, where can it go when urged by the fall of the chest-wall, but through the small side apertures between the pulmonary arteries and pulmonary veins?" Apparently Galen did not attach much importance to the blood in the pulmonary veins. It seems as if he thought it got into the veins by a sort of *side issue*. The imaginary pores in the septum of the heart appear to have been to him the great channels by which the blood reaches the left ventricle. Still, it must be admitted that Galen very nearly discovered the pulmonary circulation. His teaching with regard to the heart and pulse is also of interest, for it encumbered physiology until Harvey showed its error. Hippocrates, and Galen five centuries later, compared the heart's action to that of the chest. As the chest moves up and down, inspiring and expelling air like a pair of bellows, in like manner the heart, with its valves, acts like bellows, forcibly drawing blood into its right, and air into its left side, and then expelling both by the arteries. This indrawing of the blood and air, like the act of inspiration, was thought to be the more forcible of the two movements. Galen also regarded the pulsatile movements of arteries as a bellows movement, the artery drawing in blood and air when it expands, and expelling them when it contracts, and he thought that the expansion of the arteries is synchronous with the dilatation of the heart—a great error.

Now, that is the doctrine regarding the motion of the blood which continued to hamper the progress of medicine for many centuries, and undoubtedly the reason why Galen, with all his ingenuity, failed to correct so many errors, was that he was not sufficiently impressed with the necessity for bringing all theories to the touchstone of exact observation and thoughtfully planned experiment.

The next discoverer was Michael Servetus, the Spaniard, whose tragic death at the stake—because of his religious opinions—ended untimely a life that might have rendered much service to science. In a work on "The Restoration of Christianity," published in 1553,* Servetus states that comparatively little blood permeates the septum of the heart; most of it passes through the pulmonary artery to the lungs, where it becomes of a crimson color, and returns to the heart by the pulmonary veins, which carry air as well as blood. Some years later, Columbo, professor

* See the valuable work, "Servetus and Calvin," by R. Willis, M.D., London, 1877.

of anatomy at Padua, and Cesalpinus, of Pisa, also described the passage of the blood through the lungs.

We come now to the discoveries of Harvey.

William Harvey, a native of Folkestone, lived at a time which will be forever memorable in the history of England. It was the time of Shakspeare and Milton, of Dryden and Ben Jonson, of Bacon and Robert Boyle, and it is not too much to say that Shakspeare did not do more for English literature than did Harvey for English medicine. Harvey was well educated. He took the degree of Bachelor of Arts at the University of Cambridge, and as the medical schools of Italy were then more celebrated than those of England, he went to Padua, and there studied medicine for five years. He returned to England, took the medical degree of Cambridge, and settled in London. He was appointed physician to James I., and afterward to Charlcs I., who took a deep interest in his experiments, and gave him for the purpose of vivisection many animals from the royal parks. In 1615 Harvey was chosen to give lectures on anatomy at the London College of Physicians, and in his first course in the subsequent year he gave for the first time a public account of the results of an elaborate research "On the Motion of the Heart and Blood," which had culminated in his doctrine of the circulation. Harvey was then in his thirty-eighth year. He had been in no haste to catch the notice of the public by publishing fragments of his research, but he wisely waited until he had accumulated observations and arguments sufficient to establish his doctrine of the circulation upon a foundation that could not be shaken. You can at any time peruse the excellent translation of Harvey's works by Dr. Willis, and from them, together with the account of Harvey's life by the translator, learn how Harvey arrived at his results. He had at Padua been instructed in the doctrines of Galen. He had learned Columbo's account of the manner in which the blood passes from the right to the left side of the heart through the lungs; he had also learned the anatomy of the valves of the veins. These valves were discovered in 1754—whether by Fabricius or Sylvius is disputed—but, at any rate, they were discovered at least four years before Harvey was born. He tells us, however, that their function had been entirely misapprehended by all who had preceded him. It never occurred to them that in those veins which have valves the blood can flow only one way, and that toward the heart—the very opposite of what Galen had supposed. To that point, however, we will presently return. At the outset of his great work "On the Motion of the Heart and Blood in Animals," Harvey refers to the opinions of Galen and Columbo, and then says: "It is plain that what has heretofore been said concerning the motion and function of the heart and arteries must appear obscure, or inconsistent, or even impossible, to him who carefully considers the entire subject; it will be proper to look more narrowly into the matter; to contemplate the motion of the heart and arteries, not only in man, but in all animals that have hearts; and further, by frequent appeals to vivisection, to investigate and endeavor to find the truth."

His first concern was with the heart and arteries. He tells us that he sought by "vivisection," and not by a "study of the writings of others, to discover the motions and uses of the heart;" that he was at first greatly perplexed when he looked at the moving heart; but that by "daily diligence in vivisection" on a "variety of animals," he "thought he had obtained the truth." He describes what he saw when the chest of a living animal and the pericardium are laid open: "There is a time when the heart moves, and a time when it is motionless." The events are "more obvious in the colder animals, such as toads, frogs, serpents, fish, crabs, shrimps, snails, and shell-fish. They also become more distinct in warm-blooded animals, such as the dog, if they be attentively noted, when the heart begins to flag, to move more slowly, and as it were, to die; the movements then become slower, and the pauses longer, so that it is more easy to unravel what the motions really are, and how they are performed. In the pause, as in death, the heart is soft, flaccid, exhausted, lying as it were at rest" (Harvey, *lib. cit.*, p. 21). "After the pause the auricles contract and throw the blood into the ventricles, which being filled, then contract, and drive the blood into the arteries. The two motions—one of the auricles, the other of the ventricles—take place consecutively, but in such manner that there is a kind of harmony or rhythm between them" (*lib. cit.*, p. 31). Harvey, therefore, was the first to give an accurate description of the cardiac movements, and to show that the great motion of the heart is not its dilatation, but its contraction, and that the heart is not a pair of bellows like the chest, as Galen had thought.

Harvey was also the first to give a correct explanation of the pulse. He proved by experiment that the pulse in the arteries is not synchronous with, but succeeds, the impulse of the heart, and that the pulse is due to an expansion of the vessels by blood projected into them from the heart, and not to any inherent power of expanding like a pair of bellows. He divided an artery, and observed that blood flowed from its cardiac end with greatest force when the artery expands, and not when it contracts. A bellows-like action of the artery would have produced precisely the opposite effect (*lib. cit.*, pp. 134, 135). He performed other experiments on exposed arteries, and completely proved that it is the force of the heart's contraction which produces the pulse, by propelling at intervals a mass of blood into a system of vessels capable of passive expansion. Need I say how great a step was made in the progress of medicine when Harvey experimentally demonstrated the true cause of the pulse?

Harvey also experimentally demolished the old idea that air passes from the lungs into the pulmonary veins, to be mingled with blood in the heart and distributed through the arteries to the body generally. There is something like grim humor in his repeating one of Galen's experiments to confute Galen himself on this point. Harvey says (*lib. cit.*, p. 16): "If any one will perform Galen's experiment of dividing the trachea of a living dog, forcibly distending the lungs, and then tying the trachea securely, he will find when he has laid open the thorax abundance of air in the lungs, even to their extreme investing tunic, but none in either the pulmonary veins or left ventricle of the heart. But did the heart either attract air from the lungs, or did the lungs transmit any air to the heart in the living dog, by so much the more ought this to be the case in the experiment just referred to." Every surgeon knows well the importance of this discovery. In many of his operations he carefully guards against the entrance of air into blood-vessels, for he knows that death would inevitably follow the entrance of even a relatively small quantity of air into the veins.

Harvey also upset the doctrine that the blood passes from the right to the left ventricle through pores in the septum of the heart. He says "no such pores can be demonstrated" (*lib. cit.*, p. 17), and "why have recourse to invisible porosities * * * when there is so open a way" from the right to the left side of the heart through the pulmonary artery and pulmonary veins. But he also adduces experiments. He says (*lib. cit.*, p. 135) that if you tie the pulmonary veins in a living animal, and so prevent the blood from entering the left side of the heart, death ensues; and he gives you to understand that the results are similar to those in death from asphyxia—the right side of the heart becoming engorged, the left comparatively empty; and then he concludes that "as all these particulars have been recognized by the senses, it is manifest that the blood passes through the lungs, and not through the septum," in its course from the right to the left side of the heart, and that the right ventricle is intended to drive the blood through the blood-vessels of the lungs, and through them alone.

There was another great fact overlooked by all the predecessors of Harvey which was of fundamental importance in leading him to the idea that the blood moves in a circle. He evidently asked himself the question, How much blood does the heart transmit from the vena cava to the arteries in a given time? He found that in the dead body the left ventricle holds upwards of two ounces of blood. Then he says (*lib. cit.* p. 48), let us assume how much less the heart will hold when contracted than it does when dilated, and how much blood it projects into the aorta at each contraction. "Let us suppose that, as approaching the truth, the fourth or fifth, or sixth, or even but the eighth part of its charge, is thrown into the artery at each contraction. This would give either half an ounce, or three drachms, or one drachm, of blood as propelled by the hear at each pulse into the aorta, which quantity, by reason of the valves at the root of that vessel, can by no means return into the ventricle. Now, in the course of half an hour, the heart will have made more than one thousand beats—in some as many as two, three, and even four thousand.

Multiplying the number of drachms propelled by the number of pulses, we shall have either one thousand half-ounces or one thousand times three drachms, or a like proportional quantity of blood, according to the amount which we assume as propelled with each stroke of the heart, sent from this organ into the artery a larger quantity in every case than is contained in the whole body! * * * But let it be said that this does not take place in half an hour, but in an hour, or even in a day; any way, it is still manifest that more blood passes through the heart" (in that time) "than can either be supplied by the whole of the food consumed, or than can be contained in the veins at the same moment. * * * In short, it could be furnished in no other way than by making a circuit and returning." And then he says: "This truth indeed presents itself obviously before us when we consider what happens in the dissection of a living animal. The great artery need not be divided, but only a small branch, to get the whole blood in the body, that of the veins as well as that of the arteries, drained away in the course of half an hour or so" (*lib. cit.*, p. 50). Observe how he proved by that experiment that all the blood of the veins flows through the heart into the arteries, and can escape by an opening made into one of them, even one of small size. These are the facts that led Harvey to believe that the blood moves in a circle, and it is to be observed that he nowhere mentions the valves of the veins as in any way assisting him toward his great conception.

With regard to the flow of blood in the veins, Harvey demonstrated that the blood flows only toward the heart. He opened the jugular vein in a living animal, and found that it ceased to bleed when its cranial end was squeezed, but continued to bleed although its cardiac end was compressed, proving that the blood flows in the vein toward the chest, and not from it, as Galen supposed. He experimented on the flow in the veins of the human forearm. He tied a fillet round the upper arm, and showed that the veins on the cardiac side of it become empty, while those on the distal side become engorged; proving that the blood was prevented by the fillet from flowing through the veins toward the heart.. He observed in the bandaged arm the bead-like swellings of the distended veins that mark the situations of the valves.

He applied two fingers to a vein: with one he pressed the vein to prevent the entrance of more blood from below, while with the other he squeezed the blood out of the vein upward past a valve, and observed that the blood could not return to the emptied segment because of the valve. In that way he demonstrated the function of the venous valves.

If Harvey had only had a microscope able to reveal the capillaries in the web of a frog, in the wing of a bat, or in the tail of a fish, he could readilly have demonstrated the actual fact that the blood does flow from the arteries to the veins. But it was reserved for Malpighi to furnish that final link in the chain of actual demonstration. Harvey had to fight his battle in ignorance of the capillaries. He could only say, "The blood must pass from the arteries to the veins and return to the heart, but I cannot exactly say through what channels the passage is effected; the blood appears to pass through the pores of the tissues."

There was much commotion when Harvey proclaimed his doctrine. Many blamed him for his temerity in daring to call in question the doctrines of the ancients; many denied the truth of what he said. But he was indefatigable in demonstrating his experiments, and in devising new ones to carry his case, and he lived to see his doctrine at length accepted by nearly all. But observe how history repeats itself. Harvey, in one of his letters (*lib cit.*, p 109) says: "There are some who say that I have shown a vainglorious love of vivisection, and who scoff at the introduction of frogs and serpents, flies, and other lower animals upon the scene, as a piece of puerile levity, and they do not hesitate to use opprobrious epithets. But to return evil-speaking with evil-speaking I hold to be unworthy in a philosopher and searcher of the truth. I believe I shall do better and more advisedly if I meet so many indications of ill-breeding with the light of faithful and conclusive observation. Detractors, mummers, and writers filled with abuse, I resolved never to read them, satisfied that nothing solid or excellent, nothing but disagreeable terms, was to be expected from them, so have I held them still less worthy of an answer. Let them consume on their own ill-nature; let them go on until they are weary, if not ashamed.'

These are the words of Harvey. His detractors did at length grow weary. Even in these days of detraction he—the greatest vivisector this country has ever known—is treated with respect, and a monument is raised to his memory in his native town. How often it has happened in the history of science, that those who cannot comprehend the aims and methods of the searcher after scientific truth have been all too ready to cast a stone. So it was with one so great as Harvey. His humble followers need not wonder if they fare no better.

The work of Harvey was worthily followed up by the Reverend Stephen Hales, rector of Faringdon, in Hampshire, and minister of Teddington-on-Thames. To him we owe the first measurement of the pressure of the blood in arteries and veins. This knowledge was entirely arrived at by experiments on living animals. In his valuable "Statical Essays" (London, 1740), ordered to be printed by the Royal Society of London, he gives the details of experiments on animals, in which he introduced a long, upright glass tube into one cf the larger vessels, and observed the height of the column of blood which the pressure could support. He made many experiments on different animals; he observed the effects of various conditions on the blood-pressure, and so tapped a spring of knowledge that still flows with new discoveries. In physiology and pathology there is scarcely any subject which requires to be more frequently taken into account than the blood-pressure. It is, like the pulse, a household word to the physiologist.

I fancy the Rev. Stephen Hales would have been greatly gratified, could he have seen all that was to flow from his experiments, and I rather wonder what he would have said had he lived until now, and been asked to conduct an anti-vivisection prayer meeting.* I am sure he would have felt no little sadness at the strange short-sightedness of some of his fellow-Christians.

From Harvey and Hales let us pass to Hunter. John Hunter was a Scotchman of whom we have reason to be proud. We have, indeed, raised no monument to his memory, but he needs none. The great Museum of the Royal College of Surgeons of

* An "anti-vivisection prayer meeting," duly advertised at intervals during the past two years, has been one of the features of the anti-vivisection movement in Edinburgh.

London, one of the wonders of the scientific world, is his monument. *Si monumentum requiris, circumspice*, may truly be said by those who gaze at his work. I have no time to detail to you why Hunter has been termed the father of modern surgery, but you will find it was because he was a great physiologist. He did many experiments on living animals. The results of one of these led him to revolutionize the treatment of aneurism—that disease of arteries always so dangerous, and nearly always deadly, before he invented the operation which bears his name. The singular history of that operation has been well told by Professor Owen, formerly Curator of the Hunterian museum, and therefore one who is intimately acquainted with the history of Hunter's work.* Hunter was interested in the growth and shedding of the stag's antlers, and being curious to know what would happen to the growing antler if deprived of its supply of blood, he tied the external carotid artery on one side of the neck. On examining the antler a week later, he found, to his surprise, that its root was warm and pulsating, as it had been previous to the operation. His first impression was that he had not tied the artery properly. To satisfy himself on that point, he had the animal killed, the arteries of its head and neck injected, and then minutely dissected; and he found that the carotid had been duly tied, but that small branches springing from the cardiac side of the ligature had enlarged, and had thus carried by collateral channels blood to the antler. He had not anticipated this result. He now saw that small blood-vessels may become enlarged, to meet altered physiological circumstances. He quickly translated the thought to popliteal aneurism. He determined to tie the artery on the cardiac side of the throbbing tumor, and hoped that while the stagnant blood would coagulate within the aneurismal sac and terminate the agony of its throb, and the fatal danger of its bursting, still, haply, the limb beyond the seat of ligature might not die, but might be saved by the gradual establishment of a collateral circulation, as in the case of the antler. It was even so. The scientific instinct of Hunter guided him aright, and ever since his day the Hunterian operation for aneurism is that

---

* See *Brit. Med Jour.*, 1879, Vol. I, p. 284.

adopted; and it would be difficult to estimate the number of limbs and lives that have been saved owing to the thoughtful observation of the effect of tying an artery in the neck of a stag.

But how can I possibly recite in an hour all the discoveries regarding the circulation made by vivisection? Harvey and all before him thought that the heart is the great source of the heat of the blood. The great phenomenon of fever is an increased production of heat. How could we hope to gain a true theory of heat-production in fever if we are still under the delusion that the heart is the great source of heat? Experiments on animals alone showed that the heart is not a more important source of heat than any other muscle, and that the hottest blood of the body is not in the heart.

Our knowledge of the causes of the sounds of the heart we owe to experiments on animals, aided by the observation of diseased conditions. The late Dr. Hope, when a resident physician of the Royal Infirmary of Edinburgh, performed the celebrated experiment in which he hooked aside the sigmoid valves at the arterial openings of the heart of an animal, and found that the second cardiac sound disappeared. Also, by experiments on animals, it has been shown that the first sound of the heart depends on vibration of the auriculo-ventricular valves, and the contracting muscular fibers of the ventricles. The knowledge thus gained experimentally is daily of service to the physician. When he hears the second sound of the heart changed from a sharp click into a blowing murmur, loudest over the third right costal cartilage, he knows at once that the sigmoid valves of the aorta are no longer preventing the return of blood from the aorta into the heart, that the left side of the heart has its labor increased, and that the life of the individual is in great danger if ever he overtasks the heart by severe exertion. Many other facts of value in practical medicine attach themselves to a knowledge of the causes of the cardiac sounds.

Again, where shall we find in physiology and pathology a subject more important than the functions of the nerves of the heart and blood-vessels? How could we know that the heart has within itself the nervous apparatus essential for its movement, unless we excised the heart of an animal—a frog, for example—

and found that it still goes on beating, and that when cut into pieces, that portion containing the nerve-cells is the only part which continues to beat? How could we have known the great fact that there is a nerve which controls the cardiac movements, and can arrest them, and can in some occasion a fatal swoon, if it had not been discovered by Weber that when the pneumogastric nerve is divided and its cardiac end stimulated the heart's action is always retarded, and it may be for a time arrested? That experiment laid the foundation for our knowledge of inhibitory nervous action—a subject of far-reaching significance, and essential for a knowledge of the function of the nervous system. Again, how could we, but by experiment on animals, have made the important discovery that the nerves which inhibit the heart are affected by various drugs; some, like atropia, paralyzing them; others, like pilocarpine, increasing their sensitiveness? Again, all that we know definitely regarding nerves that accelerate the heart's action we owe to experiments on animals performed by Ludwig and others. The key to our knowledge of the great system of nerves that presides over the blood-vessels was discovered by experiment. Bernard divided the sympathetic nerve in the neck of a rabbit, and observed that the blood-vessels on the same side of the head became dilated. The cranial end of the divided nerve was afterward stimulated, and pallor of the previously congested part was the result. That was the discovery of vaso-motor nerves. All the arteries and veins in the body are governed by such nerves. Were we still ignorant of them, we could not understand the pathology of those forms of paralysis termed reflex, and, necessarily, we could not devise any rational method of treatment. And again, the nerves which cause dilatation of blood-vessels—our knowledge of such nerves we owe entirely to experiments on animals.

Time will not allow me to do more than merely say that to experiments on animals we owe much knowledge regarding the action of drugs on the heart; regarding the coagulation of the blood; regarding its respiratory changes and the manner in which its composition is affected by the inhalation of various gases; indeed, to show fully all that we have learned concerning the circulation from experiments on animals would require at least ten lectures instead of one. Other departments of physiology also teem with facts arrived at by vivisection. Were all these facts eliminated from physiology and from medicine, the remnant would indeed be a sorry figure, and in this age of brilliant scientific advancement, those who profess medicine would be treated with contempt, because of their almost complete ignorance of the bodily mechanism. I am convinced that if unbiased minds would only consider what has actually been achieved for medical science by experiments on living animals, they would not fail to see the necessity for allowing without hindrance all duly qualified medi. cal men, willing to undertake the labor, to carry on in the future that kind of research which has yielded so much fruit in the past. And although the great majority of even the educated public can not, from want of a medical education, fully understand our methods of research and the objects we have in view, yet they ought to credit us with sincerity, and with a desire to pursue in a humane manner the search for that knowledge by which the whole civilized world profits daily.—*London Lancet.*

